# ﻿*Corythoichthysquattuordecim*, a new pipefish (Teleostei, Syngnathidae) from the Coral Sea

**DOI:** 10.3897/zookeys.1244.153942

**Published:** 2025-07-08

**Authors:** Daijiro Yuki, Ronald Fricke, Hiroyuki Motomura

**Affiliations:** 1 The United Graduate School of Agricultural Sciences, Kagoshima University, 1-21-24 Korimoto, Kagoshima 890-0065, Japan Kagoshima University Kagoshima Japan; 2 Staatliches Museum für Naturkunde Stuttgart, Rosenstein 1, Gewann Rosenstein, 70191 Stuttgart, Germany Staatliches Museum für Naturkunde Stuttgart Stuttgart Germany; 3 The Kagoshima University Museum, 1-21-30 Korimoto, Kagoshima 890-0065, Japan The Kagoshima University Museum Kagoshima Japan

**Keywords:** *Corythoichthysamplexus*, morphology, New Caledonia, Pacific Ocean, taxonomy, Vanuatu

## Abstract

A new species of pipefish, *Corythoichthysquattuordecim* sp. nov., is described based on five specimens collected from Vanuatu and the Chesterfield Islands, New Caledonia. The species inhabits relatively deeper waters (56–81 m) compared to most of its congeners, which are typically found in shallower habitats. It is distinguished by a unique combination of morphological features, most notably the presence of 14 trunk rings, a rare meristic character within the genus. Additional diagnostic characters include a total of 51–52 body rings, 27 dorsal-fin rays, 5.0–5.75 subdorsal rings, and 16 or 17 pectoral-fin rays. Proportional traits include head length 14.1–17.4% of standard length, snout length 56.5–62.9% of head length, dorsal-fin base length 48.3–54.9% of head length, and snout depth 8.3–15.3% of snout length. The opercle bears a denticulate longitudinal ridge with two or three black vertical stripes, and the dorsal fin originates behind the posterior margin of anal ring. The new species most closely resembles *C.amplexus* and an undescribed taxon reported by Fricke (2004) but is clearly distinguished from both based on the above characters.

## ﻿Introduction

*Corythoichthys* Kaup, 1853 (Syngnathidae) is a genus of reef-associated pipefish comprising at least 11 recognized species (Dawson 1977; Allen and Erdmann 2008): *C.amplexus* Dawson & Randall, 1975, *C.benedetto* Allen & Erdmann, 2008, *C.flavofasciatus* (Rüppell, 1838), *C.haematopterus* (Bleeker, 1851), *C.insularis* Dawson, 1977, *C.intestinalis* (Ramsay, 1881), *C.nigripectus* Herald, 1953, *C.ocellatus* Herald, 1953, *C.paxtoni* Dawson, 1977, *C.polynotatus* Dawson, 1977, and *C.schultzi* Herald, 1953. Members of the genus are broadly distributed across the tropical Indo-Pacific, ranging from the coast of East Africa to the islands of French Polynesia. An additional, yet undescribed species of *Corythoichthys* from Vanuatu was previously reported by Fricke et al. (2011). In the present study, all *Corythoichthys* specimens held in the Muséum national d’Histoire naturelle, Paris (MNHN), were examined, revealing three individuals that correspond to this undescribed taxon from Vanuatu and the Chesterfield Islands. Furthermore, two additional specimens from the Chesterfield Islands, previously reported as *C.nigripectus* by Fricke (2004) and deposited in Staatliches Museum für Naturkunde Stuttgart, were re-examined and found to be conspecific with the MNHN specimens. These five specimens are herein described as a new species.

## ﻿Materials and methods

Counts and measurements followed Dawson (1977) and Yuki et al. (2024). Type specimens are deposited at Systématique et évolution, Laboratoire d’Ichthyologie générale et appliquée, Muséum national d’Histoire naturelle, Paris, France (**MNHN-IC**) and Staatliches Museum für Naturkunde Stuttgart, Stuttgart, Germany (**SMNS**).

## ﻿Results

### ﻿ Corythoichthys quattuordecim sp. nov.

Taxon classificationAnimaliaSyngnathiformesSyngnathidae

684DBCF8-215F-571B-A8C1-76DB44A0B503

https://zoobank.org/4E26D87A-84C0-4632-9A52-31DC3DADECED

**Figure 1.**
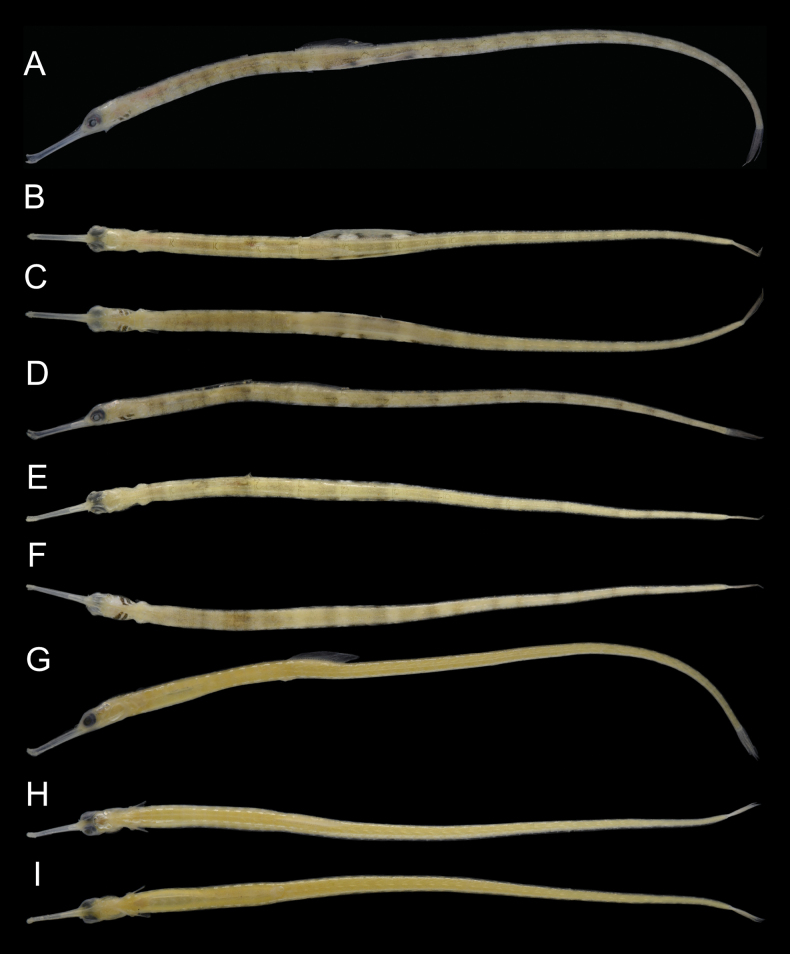
Preserved specimens of *Corythoichthysquattuordecim* sp. nov. **A–C.**MNHN-IC-2010-0773, male, 68.3 mm SL; **D–F.**MNHN-IC-2010-0774, male, 57.4 mm SL; **G–I.**MNHN-IC-2004-2018, female, 80.2 mm SL. **A, D, G.** Lateral views; **B, E, H.** Dorsal views; **C, F, I.** Ventral views.

Fig. 1 ; Table 1 New English name: Coral Sea Pipefish

Corythoichthys nigripectus : Fricke 2004: 16 (Chesterfield Islands, New Caledonia).
Corythoichthys sp.: Fricke et al. 2011: 388 (Vanuatu).

#### Types.

***Holotype*.** • MNHN-IC-2010-0773, 68.3 mm SL, off Mavéa Island, Santo, Vanuatu, 15°22'22.8"S, 167°12'36"E, 57–81 m depth, 27 Sept. 2006, RV *Alis*, Campagne Santo 6, St. at39.

***Paratypes*.** • 4 specimens: MNHN-IC-2004-2018, 80.2 mm SL, Chesterfield Islands, New Caledonia, 19°25'00"S, 158°24'36"E, 56 m depth, 28 July 1988, RV *Coriolis*, Campagne Corail 2, St. DW119; • MNHN-IC-2010-0774, 57.4 mm SL, collected with holotype; SMNS 21754, 86.8 mm SL, collected with the holotype; • SMNS 21773, 70.0 mm SL, northern lagoon, west of Île Squeleton, Chesterfield Islands, New Caledonia, 19°17'32"S, 158°34'09"E, 65–68 m depth, 18 July 1984, RV *Coriolis*, Campagne Chalcal 84, St. CP 7.

#### Diagnosis.

Trunk rings 14; total rings 51 or 52; pectoral fin rays 16 or 17 usually 16; dorsal fin origin behind posterior margin of anal ring; opercle with a denticulate longitudinal ridge; opercle with two or three black lines; dorsal fin without rows of pale spots; venter of anterior trunk rings without blotches, spots, or lines; venter of anal ring without black blotch.

#### Description.

Counts and measurements listed in Table 1. Body elongated, slightly compressed; body transverse section trapezoidal, dorsal section shorter than ventral section; trunk shorter than tail; first trunk ring longer than second; superior ridges entirely to minutely denticulate; superior trunk ridges indented to distinctly notched between rings, usually elevated well above level of dorsum; no dermal flaps or keeled scutella present, principal body ridges distinct; superior trunk and tail ridges discontinuous; lateral trunk ridge straight, end tips of anal ring; inferior trunk and tail ridges continuous; snout slender, with low, denticulate, median dorsal ridge but without median lateral ridge, spines or spinules; eye slightly prominent, orbital margin not expanded laterally above or below; supraorbital and paired interorbital ridges denticulate, distinct; opercle with a denticulate longitudinal ridge; supraopercular ridge short, inconspicuous; pectoral-fin base with two distinct ridges, lower ridge denticulate. Dorsal-fin base slightly raised; posterior margin of pectoral fin rounded; anal fin very small; caudal fin rounded, membranes between each soft ray with slit. Brood pouch on ventral surface from first to 16^th^ tail rings in males, with fleshy folds but lacking protective plates.

**Table 1.** Counts and measurements of *Corythoichthysquattuordecim* sp. nov.

	Holotype	Paratypes			
	MNHN-IC-2010-0773	MNHN-IC-2004-2018	MNHN-IC-2010-0774	SMNS 21754	SMNS 21773
Standard length (mm; SL)	68.3	80.2	57.4	86.8	70.0
Sex	male	female	male	male	female
Counts					
Dorsal-fin rays	27	27	27	27	27
Pectoral-fin rays (left/right)	16/16	16/16	16/17	17/17	16/16
Anal-fin rays	4	4	4	4	4
Caudal-fin rays	10	10	10	10	10
Subdorsal rings	5	5.75	5.25	5.5	5.25
Subdorsal trunk rings	0.5	0.5	1.0	0.5	0.5
Trunk rings	14	14	14	14	14
Tail rings	37	38	37	37	37
Measurements (% SL)					
Head length (HL)	14.9	14.1	15.9	14.6	17.4
Trunk length	21.6	22.4	21.8	21.9	35.4
Tail length	62.1	62.5	61.7	63.9	53.6
Snout length	8.6	8.0	9.4	9.2	10.3
Snout depth	0.8	5.2	0.8	1.2	1.6
Orbit diameter	2.7	2.7	2.9	2.5	2.7
Postorbital length	3.9	3.8	3.9	3.3	3.9
Distance from posterior margin of orbit to central pectoral-fin base (DPP)	5.2	5.2	5.5	4.6	6.1
Head width	3.5	3.6	3.4	3.1	3.6
Body depth	2.7	2.5	2.4	2.4	3.0
Dorsal-fin base length	7.6	7.4	7.7	8.0	7.9
Pectoral-fin base length	1.4	1.5	1.4	1.3	1.6
Measurements (% HL)					
Snout length	57.5	56.5	59.0	62.9	59.0
Snout depth	5.6	5.8	4.9	7.8	9.0
Orbit diameter	18.2	19.4	18.5	18.1	15.6
Postorbital length	25.9	26.9	24.6	22.8	22.1
DPP	35.1	36.9	34.3	31.4	35.2
Head width	23.1	25.5	21.6	21.2	20.5
Body depth	17.9	17.6	15.1	16.5	19.1
Dorsal-fin base length	51.2	52.4	48.3	53.1	54.9
Pectoral-fin base length	9.2	10.7	8.8	8.7	10.7
Measurements (% snout length)					
Snout depth	9.7	10.3	8.3	12.5	15.3
DPP	61.0	65.3	58.1	53.0	59.7

***Color of preserved specimens*** (Fig. 1). Interorbital and dorsum of head without stripes or somewhat reticulate pattern of black or dark brown lines; melanophores scattered; median dorsal snout ridges without dark brown spots. Side of snout with melanophores on posterior one-third; dorsum transparent with melanophores or a single spot on either side of median ridge; postorbital and opercle above opercular ridge with melanophores, below opercular ridge with two or three vertical stripes extending ventrally. Dorsum and sides of trunk and tail with indistinct reticulate bands and scattered melanophores; lower part of trunk without 1–3 irregular rows of small brown spots; first trunk ring with small number of melanophores ventrally, without stripes, blotches, bars, or shaded or lined pattern; second to 14^th^ trunk rings with melanophores ventrally. First to 20^th^ tail rings with many melanophores ventrally; behind 21^st^ tail ring, a low number of melanophores ventrally. Dorsal, anal, and caudal fins with melanophores. Pectoral fins transparent. A faded specimen (MNHN-IC-2004-2018) lacked all melanophores.

#### Distribution.

Currently known only from Vanuatu and New Caledonia.

#### Etymology.

The specific name ‘‘*quattuordecim*’’, derived from Latin, refers to the 14 trunk rings in the new species.

#### Remarks.

The new species was identified as belonging to *Corythoichthys* based on the diagnosis for the genus, given its possession of superior trunk and discontinuous tail ridges; inferior trunk and continuous tail ridges; lateral trunk ridge discontinuous near the end of the anal ring; dorsum somewhat depressed between superior body ridges; dorsal-fin base not distinctly elevated; opercle with low longitudinal keel; a brood pouch under the tail; trunk rings 14; tail rings 37 or 38; subdorsal rings 5.0–5.75, dorsal-fin rays 27; pectoral-fin rays 16 or 17; anal-fin rays 4; and caudal-fin rays 10 (Dawson 1977, 1985).

*Corythoichthysquattuordecim* possesses 14 trunk rings, whereas the modal trunk ring count of *Corythoichthys* is 15–18 (Dawson 1977; Fricke 2004; Allen and Erdmann 2008; Yuki et al. 2024) [but 14 trunk rings in *C.amplexus* and *Corythoichthys* sp. sensu Fricke, 2004 (Dawson 1977; Fricke 2004; Allen and Erdmann 2008; Yuki et al. 2024)]. *Corythoichthysquattuordecim* is clearly distinguished from *C.amplexus* in having 16 or 17 pectoral-fin rays (vs 12–15 in *C.amplexus*), the snout length 56.5–62.9% HL (35.7–47.6%), snout depth 8.3–15.3% snout length (18.5–27.0%), and body without broad dark bands crossing the sides and dorsum (with such broad dark bands, sometimes divided to form two close-set bands) (Dawson 1977, 1985; Allen and Erdmann 2008). *Corythoichthysquattuordecim* is distinguished from *Corythoichthys* sp. sensu Fricke, 2004 in having the head length 14.1–17.4% SL (vs 9.7–10.6%), body depth 2.4–3.0% SL (vs 3.0–3.4%), snout length 56.5–62.9% HL (vs 35.7–38.5%), 5.0–5.75 subdorsal rings (vs 6.0–7.0), the opercle with a denticulate longitudinal ridge (vs longitudinal ridge not denticulate), and body without broad dark brown bars (vs with broad dark-brown bars) (Fricke 2004). The new species is similar to *C.ocellatus* and *C.schultzi* in snout length (56.5–62.9% of head length in *C.quattuordecim*, 50.0–58.8% in *C.ocellatus*, 50.0–66.7% in *C.schultzi*) (Dawson 1985), but it is distinguished from both species in having 14 trunk rings (15 or 16 in *C.ocellatus*, 15–17 in *C.schultzi*), 27 dorsal-fin rays (22–25 in *C.ocellatus*), 37 or 38 tail rings (29–32 in *C.ocellatus*), and the opercle with a denticulate longitudinal ridge (longitudinal ridge not denticulate in either) (Dawson 1985).

Members of *Corythoichthys* typically inhabits waters shallower than 40 m depth (Dawson 1985), although the deepest recorded occurrence is at 125–128 m for *C.nigripectus* (Fricke 2004). Collected at depths of 56–81 m, *C.quattuordecim* occupies relatively deeper habitats compared to most congeners. The paratypes (MNHN-IC-2004-2018, SMNS 21754, and SMNS 21773) were previously reported as *C.nigripectus* by Fricke (2004, in part, Chesterfield Islands).

## Supplementary Material

XML Treatment for Corythoichthys quattuordecim

## ﻿Additional information

### Use of AI

No use of AI was reported.

### Author ORCIDs

Daijiro Yuki https://orcid.org/0009-0003-2367-741X

Ronald Fricke https://orcid.org/0000-0003-1476-6990

Hiroyuki Motomura https://orcid.org/0000-0002-7448-2482

### Data availability

Data generated during and/or analyzed in the present study are available in this published article.

## References

[B1] Allen GR Erdmann MV (2008) *Corythoichthysbenedetto*, a new pipefish (Pisces: Syngnathidae) from Indonesia and Papua New Guinea. Aqua. International Journal of Ichthyology 13: 121–126.

[B2] Dawson CE (1977) Review of the pipefish genus *Corythoichthys* with description of three new species. Copeia 1977(2): 295–338. 10.2307/1443912

[B3] Dawson CE (1985) Indo-Pacific Pipefishes (Red Sea to the Americas). The Gulf Coast Research Laboratory, Ocean Springs, [vi +] 230 pp.

[B4] Fricke R (2004) Review of the pipefishes and seahorses (Teleostei: Syngnathidae) of New Caledonia, with descriptions of five new species. Stuttgarter Beiträge zur Naturkunde, Serie A, Biologie 668: 1–66.

[B5] Fricke R Kulbicki M Wantiez L (2011) Checklist of the fishes of New Caledonia, and their distribution in the Southwest Pacific Ocean (Pisces). Stuttgarter Beiträge zur Naturkunde A, Neue Serie 4: 341–463.

[B6] Yuki D Endo H Motomura H (2024) First Japanese record of *Corythoichthysintestinalis* (Teleostei: Syngnathidae) from the Ryukyu Islands. Species Diversity: An International Journal for Taxonomy, Systematics, Speciation, Biogeography, and Life History Research of Animals 29(2): 247–253. 10.12782/specdiv.29.247

